# Biochemical and structural characterization of a DNA N^6^-adenine methyltransferase from *Helicobacter pylori*

**DOI:** 10.18632/oncotarget.9692

**Published:** 2016-05-29

**Authors:** Bo Ma, Ji Ma, Dong Liu, Ling Guo, Huiling Chen, Jingjin Ding, Wei Liu, Hongquan Zhang

**Affiliations:** ^1^ Department of Human Anatomy, Histology and Embryology, Key Laboratory of Carcinogenesis and Translational Research, Ministry of Education, and State Key Laboratory of Natural and Biomimetic Drugs, Peking University Health Science Center, Beijing, China; ^2^ Institute of Immunology, The Third Military Medical University, Chongqing, China; ^3^ Institute of Biophysics, Chinese Academy of Sciences, Beijing, China

**Keywords:** DNA N6-adenine methyltransferase, M1.HpyAVI, substrate recognition, AdoMet-binding, Helicobacter pylori, Immunology and Microbiology Section, Immune response, Immunity

## Abstract

DNA N^6^-methyladenine modification plays an important role in regulating a variety of biological functions in bacteria. However, the mechanism of sequence-specific recognition in N^6^-methyladenine modification remains elusive. M1.HpyAVI, a DNA N^6^-adenine methyltransferase from *Helicobacter pylori*, shows more promiscuous substrate specificity than other enzymes. Here, we present the crystal structures of cofactor-free and AdoMet-bound structures of this enzyme, which were determined at resolutions of 3.0 Å and 3.1 Å, respectively. The core structure of M1.HpyAVI resembles the canonical AdoMet-dependent MTase fold, while the putative DNA binding regions considerably differ from those of the other MTases, which may account for the substrate promiscuity of this enzyme. Site-directed mutagenesis experiments identified residues D29 and E216 as crucial amino acids for cofactor binding and the methyl transfer activity of the enzyme, while P41, located in a highly flexible loop, playing a determinant role for substrate specificity. Taken together, our data revealed the structural basis underlying DNA N^6^-adenine methyltransferase substrate promiscuity.

## INTRODUCTION

DNA methylation is a common form of modification on nucleic acids occurring in both prokaryotes and eukaryotes. Such a modification creates a signature motif recognized by DNA-interacting proteins and functions as a mechanism to regulate gene expression [1]. DNA methylation is mediated by DNA methyltransferases (MTases), which catalyze the transfer of a methyl group from S-adenosyl-L- methionine (AdoMet) to a given position of a particular DNA base within a specific DNA sequence [2].

Three classes of DNA MTases have been identified to transfer a methyl group to different positions of DNA bases. C^5^-cytosine MTases, for example, methylate C^5^ of cytosine (m5C). In eukaryotes, m5C plays an important role in gene expression, chromatin organization, genome maintenance and parental imprinting, and is involved in a variety of human diseases including cancer [3]. By contrast, the functions of the prokaryotic DNA cytosine MTase remain unknown. N^4^-cytosine MTases, which are frequently present in thermophilic or mesophilic bacteria, transfer a methyl group to the exocyclic amino group of cytosine (4mC) [4]. N^4^ methylation seems to be primarily a component of bacterial immune system against invasion by foreign DNA, such as conjugative plasmids and bacteriophages [5]. The third group, N^6^-adenine MTases methylate the exocyclic amino groups of adenine (6mA), which exists in prokaryotes as a signal for genome defense, DNA replication and repair, regulation of gene expression, control of transposition and host-pathogen interactions [3]. Recent studies utilizing new sequencing approaches have showed the existence of 6mA in several eukaryotic species [6, 7]. DNA 6mA modification is associated with important biological processes including nucleosome distribution close to the transcription start sites in *Chlamydomonas* [8], carrying heritable epigenetic information in *C.elegans* [9] or controlling development of *Drosophila* [10].

All the three types of methylation exist in prokaryotes, and most DNA MTases are components of the restriction-modification (R-M) systems. The R-M systems are composed of two enzymes displaying opposing activities. “R” stands for a restriction endonuclease cleaving specific DNA sequences, while “M” symbolizes a modification methyltransferase rendering these sequences resistant to cleavage [11]. The cooperation of these two enzymes provides a defensive mechanism to protect bacteria from infection by bacteriophages. The R-M systems are classified into three types based on specific structural features, position of DNA cleavage and cofactor requirements. In types I and III, the DNA adenine or cytosine methyltransferase is part of a multi-subunit enzyme that catalyzes both restriction and modification. By contrast, two separate enzymes exist in type II systems, where a restriction endonuclease and a DNA adenine or cytosine methyltransferase recognize the same targets [3].

To date, a number of bacterial DNA MTases have been structurally characterized, covering enzymes from all the three classes. All these MTases exhibit high similarity in their overall architectures, which are generally folded into two domains: a conserved larger catalytic domain comprising an active site for methyl transfer and a site for AdoMet-binding, and a smaller target (DNA)-recognition domain (TRD) containing variable regions implicated in sequence-specific DNA recognition and the infiltration of the DNA to flip the target base [12]. Conserved amino acid motifs have been identified from reported structures, including ten motifs (I-X) in cytosine MTases and nine motifs (I-VIII and X) in adenine MTases, all of which are arranged in an almost constant order. According to the linear arrangement of three conserved domains, exocyclic amino MTases are subdivided into six groups (namely α, β, γ, ζ, δ and ε). N^6^-adenine and N^4^-cytosine MTases, in particular, are closely related by sharing common structural features [12, 13]. Despite the considerable similarity among bacterial MTases, some differences were observed among the enzymes from various species. For example, the structural regions of MTases beyond the catalytic domain are rather variable, such as the C-terminal domain of M.TaqI, the extended arm of M.MboIIA and M.RsrI, the helix bundle of EcoDam, and so on [2, 14–16].

DNA methylation is thought to influence bacterial virulence. DNA adenine methyltransferase has been shown to play a crucial role in colonization of deep tissue sites in *Salmonella typhimurium* and *Aeromonas hydrophila* [17, 18]. Importantly, DNA adenine methylation is a global regulator of genes expressed during infection and inhibitors of DNA adenine methylation are likely to have a broad antimicrobial action. Dam was considered a promising target for antimicrobial drug development [18].

*Helicobacter pylori* is a Gram-negative bacterium that persistently colonizes in human stomach worldwide. It is a major pathogen of gastritis and peptic ulcer diseases as well as a cancer-causing factor for gastric cancer. *H. pylori* is involved in 90% of all gastric malignancies, infecting nearly 50% of the world's population and is the most crucial etiologic agent for gastric adenocarcinoma [19]. *H. pylori* strains possess a few R-M systems like other bacteria to function as defensive systems. *H. pylori* 26695, for example, has 23 R-M systems [20]. Methyltransferases were suggested to be involved in *H. pylori* pathogenicity [33]. M1.HpyAVI is a DNA adenine MTase that belongs to the type II R-M system. This system contains two DNA MTases named M1.HpyAVI and M2.HpyAVI, and a putative restriction enzyme. M1.HpyAVI encoded by ORF *hp0050* is an N^6^-adenine methyltransferase belonging to the β-class MTase. It has been reported recently that this enzyme recognizes the sequence of 5′-GAGG-3′, 5′-GGAG-3′ or 5′-GAAG-3′ and methylates adenines in these sequences. Given that methylation of two adjacent adenines on the same strand have never been observed for other N^6^-adenine MTases, the methylation activity on 5′-GAAG-3′ seems to be a unique feature of M1.HpyAVI, compared with the homologs from other strains of *H.pylori* which is able to methylate only 5′-GAGG-3′. The structural basis and the catalytic mechanism underlying such a distinct activity are not well understood due to the lack of an available 3D structure of this enzyme.Here, we report the crystal structure of M1.HpyAVI from *H. pylori* 26695, which is the first determined N^6^-adenine MTase structure in *H. pylori*. The structure reveals a similar architecture as the canonical fold of homologous proteins, but displays several differences in the loop regions and TRD. Based on structural and biochemical analyses, we then identified two conserved amino acids, D29 at the catalytic site and E216 close to the C-terminus, as crucial residues for cofactor binding and methyltransferase activity of M1.HpyAVI. In addition, a non-conserved amino acid, P41, seems to play a key role in substrate recognition.

## RESULTS

### Overall structure

Recombinant full-length M1.HpyAVI was produced as a soluble protein in *Escherichia coli*, but was quite unstable and tended to aggregate in low salt environment. The protein, however, remained fully soluble in a buffer containing higher concentration of sodium chloride (>300 mM), which prompted that M1.HpyAVI is likely a halophilic protein.

The cofactor-free and AdoMet-bound proteins were crystallized at different conditions. Both structures were determined by means of molecular replacement, and refined to 3.0 Å and 3.1 Å, respectively. Statistics of X-ray data collection and structure refinement were summarized in Table 1.

**Table 1:** Data collection and structure refinement statistics of M1.HpyAVI

	M1.HpyAVI	M1.HpyAVI-AdoMet complex
**Data collection**		
Wavelength (Å)	1.0000	0.97772
Space group	*P*4_3_2_1_2	*P*6_5_
Unit-cell parameters (Å, ˚)	*a* = *b* = 69.73, *c* = 532.75 *α = β = γ* = 90	*a = b* = 135.60, *c* = 265.15 *α = β* = 90, *γ* = 120
Resolution range (Å) ^a^	49.09-3.00 (3.09-3.00)	48.91-3.10 (3.18-3.10)
Unique reflections ^a^	27243	49833
Multiplicity ^a^	3.7 (3.8)	5.6 (4.0)
Completeness (%)^a^	98.7 (98.9)	99.7 (97.8)
Mean *I/δ* (*I*) ^a^	12.1 (3.4)	14.0 (1.9)
Solvent content (%)	58.67	61.96
*R*_merge_ ^a^	0.073 (0.378)	0.106 (0.769)
**Structure refinement**		
*R*_work_	0.251	0.221
*R*_free_	0.308	0.276
R.m.s.d., bond lengths (Å)	0.007	0.007
R.m.s.d., bond angles (˚)	1.408	1.651
**Ramachandran plot**		
Favoured region (%)	89.44	91.44
Allowed region (%)	9.58	7.11
Outliers (%)	0.99	1.45

a Values in parentheses are statistics of the highest resolution shell.

Four and eight protein monomers resided in the asymmetric units of the two crystal structures. Some amino acids, particularly those within two loops (residues 32-61 and 152-172) in both structures, were poorly defined in electron density and had to be omitted from the refined models. Details of invisible amino acids are given in Table S1.

**Figure 1:**
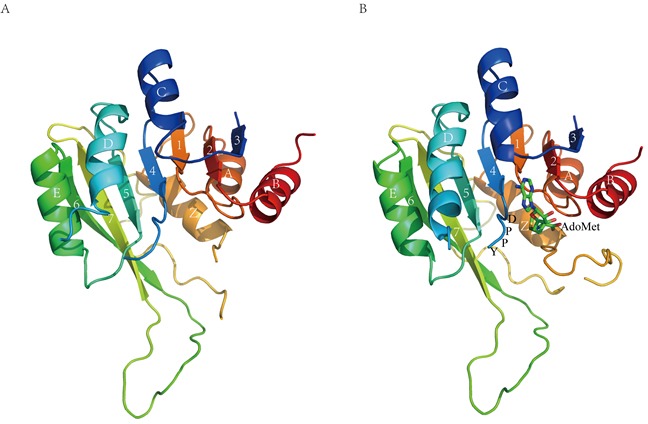
Overall structure of M1.HpyAVI **A.** Free form **B.** AdoMet-bound form. Ribbon diagram of M1.HpyAVI resembles an “AdoMet-dependent MTase fold”, a mixed seven-stranded β-sheet flanked by six α-helices, αA, αB, αZ on one side and αD, αE, αC on the other side, the cofactor AdoMet is bound in a cavity near the conserved enzyme activity motif DPPY. Rainbow coloring from blue through green to red indicates the N- to C-terminal position of the residues in the model. The α-helices and β-strands are labelled and numbered according to the commonly numbering rule for the known MTases. The AdoMet molecule is shown in green.

The two structures are very similar to each other (Figure 1) and could be well overlaid with an RMSD of 0.76 Å on 191 C_α_ atoms. The overall architecture of M1.HpyAVI revealed in these structures resembles the AdoMet-dependent MTase fold in which a twisted seven-stranded β-sheet flanked by six α-helices forms the structural core. Like the reported structures of the larger domain of MTases, three helices (αA, αB and αZ) are located at one face of the central β-sheet, while the other three αD, αE and αC sit at the other side. All these conserved structural motifs form a typical α/β Rossmann fold. The catalytic motif DPPY lies in a loop connecting αD and β4, and the cofactor AdoMet binds in a neighboring cavity. The loop (residues 136-166) located between β7 and αZ corresponds to a highly diverse region in other MTases that is involved in target DNA recognition. The hairpin loop (residues 101-133) bridging β6 and β7, which is proposed to bind DNA in the minor groove, displays a similar conformation as those observed in M.MboIIA, M.RsrI and M.pvuII [2, 5, 21]. The missing loop (residues 33-58) in the structure of M1.HpyAVI corresponds to loop I in M.TaqI, which was also invisible in a structure without DNA. This loop, however, was well ordered in an M.TaqI-DNA complex structure and was shown to play a crucial role in DNA methylation by contacting the flipping adenine and recognizing specific DNA sequence [14].

### Dimeric state of M1.HpyAVI in crystal and solution

Previous studies showed that some DNA MTases, e.g. M.BamHI and M.EcoRI [22, 23], exist as monomer in solution, in agreement with the fact that a DNA substrate for a typical MTase is hemimethylated and therefore needs only a single methylation event to convert it into a fully methylated state [12]. Increasing number of dimeric DNA MTases, however, has been identified from later studies. For instance, M.DpnII, M.RsrI, M.KpnI, and M.MboIIA have been found as dimers in solution [12, 24]. In addition, several MTases including M.MboIIA, M.RsrI and TTH0409 form tightly associated dimers in crystal structures [2, 15, 25]. Nonetheless, some DNA MTases such as M.CcrMI and the *Bacillus amyloliquefaciens* MTase dissociate from dimer into monomer upon DNA-binding [2].

**Figure 2:**
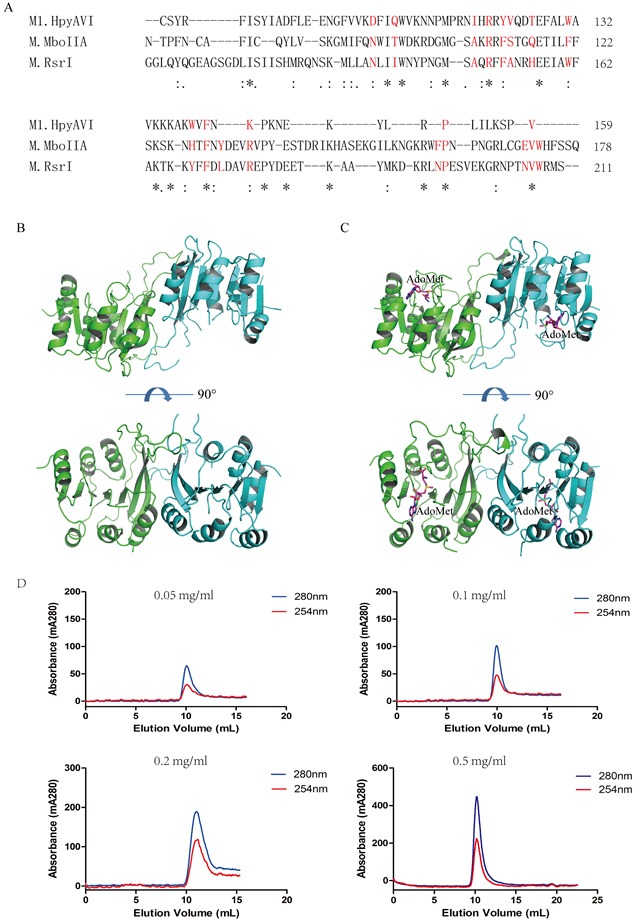
M1.HpyAVI exists as dimer in crystal and solution **A.** A conserved interface area of β-class MTases is defined in M1.HpyAVI. Residues that involved are signed in red color; Dimerization of free-form M1.HpyAVI **B.** and cofactor-bound M1.HpyAVI **C.** The two monomers are marked in green and blue, AdoMet molecules are marked in magenta. **D.** Gel-filtration analysis revealed that M1.HpyAVI exist as a dimer in solution. FPLC system coupled to a Superdex 75 10/300 column. Elution profiles at 280 nm (blue) and 260 nm (red) are: different concentration (0.05, 0.1, 0.2, 0.5 mg/ml) of M1.HpyAVI protein.

According to the arrangement of the three conserved domains, M1.HpyAVI belongs to the β-subgroup, in which a conserved motif NXXTX_9−11_AXRXFSXXHX_4_WX_6−9_ YXFXLX_3_RX_9−26_NPX_1−6_NVWX_29−34_A has been identified at the dimerization interface in crystal structures [12]. Most of conserved amino acids within that motif are present in the sequence of M1.HpyAVI (Figure 2A), implying dimerization of this protein. In agreement, a dimer of M1.HpyAVI was observed in our crystal structures with the two monomers related by a two-fold axis (Figure 2B and 2C). An area of ~1900 Å^2^ was buried at the dimeric interface, taking up ca 17% of the total area. The dimeric architecture was greatly stabilized by hydrogen bonds and salt bridges formed among residues R86, D93 and E96. In addition, comparison of the dimer structure of M1.HpyAVI with some other β-class MTases (M1.MboIIA, M.RsrI and TTHA0409) suggested that the M1.HpyAVI dimer organized in a similar form as others (Figure S3).

To probe the oligomeric form of M1.HpyAVI in solution, different concentrations of purified enzyme was loaded onto a Superdex 75 10/300 column. The protein was eluted at ~10 ml regardless of the protein concentrations, corresponding to a dimeric molecular mass of 54 kDa (Figure 2D).

Our results clearly showed that M1.HpyAVI forms a dimer in both crystal and solution as other β-class MTases, which however disagrees with a previous investigation using dynamic light scattering (DLS) measurement and gel-filtration chromatography, suggesting that M1.HpyAVI is taking a monomeric state in solution [20]. This variance might be caused by an addition of 100 mM arginine before cell lysis to keep protein solubility and also by later replacement of arginine with 30% glycerol by dialysis. These treatments probably changed protein conformation somehow and also the oligomeric state.

### Structure comparisons

**Figure 3:**
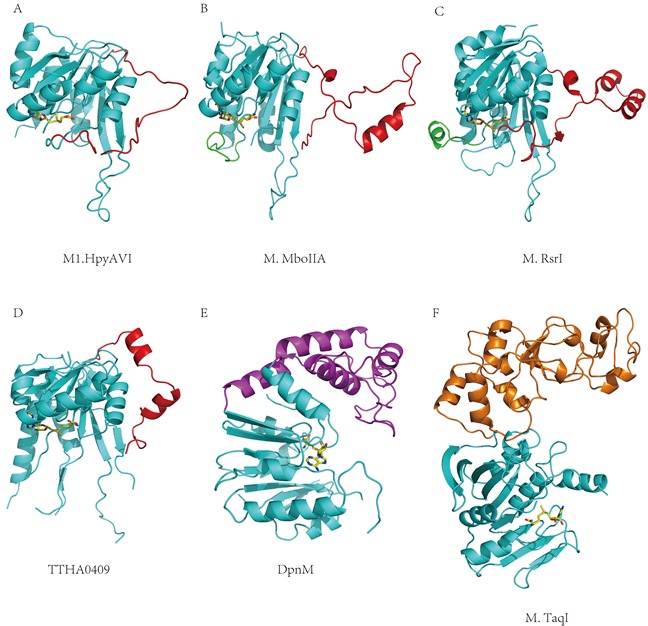
Structural comparisons between M1.HpyAVI and other DNA MTases **A.** M1.HpyAVI; **B.** M.MboIIA; **C.** M.RsrI; **D.** TTHA0409; **E.** DpnM; **F.** M.TaqI. M1.HpyAVI possesses only a long disorder TRD region, compared with the structure-rich TRD of M.MboIIA, M.RsrI and TTHA0409, or the extra DNA-binding domain of DpnM and M.TaqI. The core structure is in cyan; TRD of M1.HpyAVI, M.MboIIA, M.RsrI and TTHA0409 is in red; The region between β4 and αD of M.MboIIA and M.RsrI is in green; DNA-binding domain of DpnM is in magenta; The C-terminal domain of M.TaqI is in orange.

As a β-class N^6^ adenine MTase, the M1.HpyAVI structure displayed a good similarity with M.MboIIA (PDB ID 1G60) and M.RsrI (PDB ID 1NW7), which are falling into the same subgroup. Superimposition of M1.HpyAVI onto them gave RMSDs of 1.63 Å and 1.9 Å on 168 and 190 C_α_ atoms, respectively. The most striking structural difference was found to locate on the TRD region (residues 133-163 in M1.HpyAVI) (Figure 3A–3C), where the secondary structures vary among these structures. By comparison with the other two enzymes that possess protruding arms containing several α-helices and/or β-strands, the TRD of M1.HpyAVI is much shorter in length (Figure S1), wrapping more closely around the structural core and lacking apparent secondary structures. Given the proposed role of the TRD for DNA interaction at the major groove [2], some differences of DNA recognition mode can be expected. Another difference locates at the highly flexible loop between β4 and αD (residues 33-58) of M1.HpyAVI, which was invisible in our structures but present in the structures of M.MboIIA and M.RsrI [2, 15]. Sequence alignment revealed that this region of M1.HpyAVI was longer than its counterparts by 13 and 16 amino acids respectively, which likely renders the *H. pylori* enzyme more flexible.

N^6^-adenine and N^4^-cytosine MTases, in particular, are closely related by sharing common structural features [13]. Structural comparison between M1.HpyAVI and a putative β-class N^4^ cytosine MTase named TTHA0409 (PDB ID 2ZIF) [25] showed a good similarity as well, giving an RMSD of 1.73 Å on 164 C_α_ atoms (Figure 3D). Exactly like the above comparison, the most significant difference exists in the TRD, where the structures vary in terms of length and presence of α-helices (Figure S1).

M1.HpyAVI displayed a considerable structural dissimilarity in comparison with N^6^-adenine MTases from other subgroups including the α-class DpnM (PDB ID 2DPM) and the γ-class M.TaqI (PDB ID 2ADM). Both comparisons gave RMSDs above 3.0 Å (Figure 3E and 3F). These two enzymes lack a counterpart loop present in the TRD of M1.HpyAVI, but instead rely on an extra domain for DNA binding and sequence recognition [14, 26].

Collectively, M1.HpyAVI possesses a long disordered TRD, which is in sharp contrast to the secondary structure-rich TRD in other β-class N^6^ adenine or N^4^ cytosine MTases or the extra DNA binding domain present in DNA MTases from other subgroups. This striking difference may be a significant determinant of the wider substrate spectrum of this *H. pylori* enzyme.

### AdoMet-binding pocket

**Figure 4:**
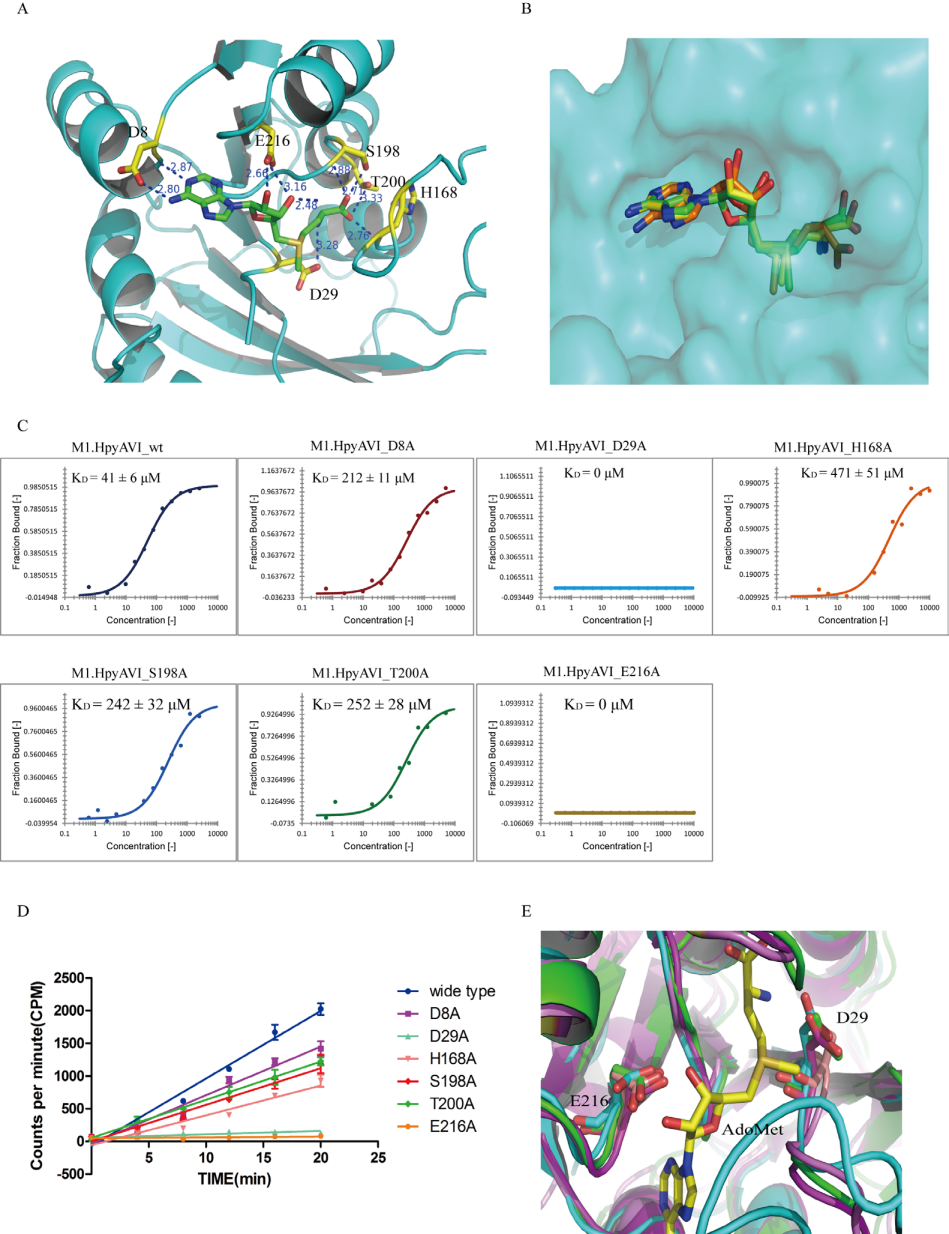
Structural and biochemical analyses define two conserved residues D29 and E216 to be the key sites for AdoMet binding **A.** The cofactor-binding cavity of M1.HpyAVI. Residues (yellow) that form direct hydrogen bonds with AdoMet (green) are indicated, distance of the hydrogen bond is marked. **B.** Superposition of AdoMet in the structures of M1.HpyAVI (green), DpnM (yellow) and M.TaqI (orange). The AdoMet terminal carboxyl of M.TaqI reveals different orientations. **C.** Cofactor binding affinity of wt-/mutants M1.HpyAVI proteins analyzed by microscale thermophoresis (MST). The binding affinity was determined between fluorescently labelled M1.HpyAVI protein and unlabeled AdoMet. The bound fraction is shown on the *y*-axis against the protein concentration. AdoMet (15 nM to 1 mM) was titrated into a fixed concentration of M1.HpyAVI wt/mutant proteins (800 nM). The dissociation constant (K_D_) is yielded according to the law of mass action from the isotherm derived of the raw data: M1.HpyAVI-wt: 41 ± 6 μM; M1.HpyAVI-D8A :212 ± 11 μM; M1.HpyAVI-D29A : 0 μM; M1.HpyAVI-H168A : 471 ± 51 μM; M1.HpyAVI-S198A : 242 ± 32 μM; M1.HpyAVI-T200A : 252 ± 28 μM; M1.HpyAVI-E216A : 0 μM. Standard for three replicates is indicated. Measurements were made with 40% LED and 40% laser power at 25°C. **D.** DNA methyltransferase activity of wide type protein and the mutants is quantified using radioactive assay. [^3^H]-methyl transferred to duplex DNA containing 5′-GAGG-3′ was quantified by Beckman LS6500 for 10 min, experiments were repeated for three times and data were corrected by subtraction of the background. **E.** Superposition of M1.HpyAVI (green) with M.MboIIA (cyan) and M.RsrI (magenta). Residues D29 and E216 are conserved through all the DNA MTases mentioned in Figure 3 (not shown in Figure 4).

The cofactor binding pocket of M1.HpyAVI is surrounded by residues 7-9, 29-31, 165-167, 216-218 and 221 (Figure 4A), which are conserved among most of DNA MTases [2]. A hydrogen bond between D29 in the catalytic motif DPPY and the amino group of bound AdoMet is preserved as other MTase structures. Residues D8 and A9 from hydrogen-bonds with N^6^ and N^1^ of the purine ring, respectively, and E216 also locates at hydrogen bonding distance with O2′ and O3′ of the ribose. In addition, H168, T200 and S198 contact the terminal carboxyl of AdoMet. Superposition of M1.HpyAVI with the five structures shown in Figure 3 reveals that the orientation of cofactor is rather conserved except for M.TaqI (Figure 4B). The different conformation of the bound cofactor observed in M.TaqI might be attributable to the absence of corresponding residues of the conserved AdoMet-binding motif FXGXG in that structure.

To confirm the key residues for ligand binding, we prepared a series of single mutants by replacing D8, D29, H168, S198, T200, E216 with alanine and investigated their ligand binding affinity using microscale thermophoresis (MST) assay. As shown in Figure 4C, by contrast to the wild type enzyme, most mutants displayed variable reduction of K_D_ value, among them the D29A and E216A mutants displayed no protein-AdoMet affinity at all.

The results suggested that the hydrogen bonds formed by D29 and E216 with AdoMet were most crucial interactions for cofactor binding. Mutation of the two residues may directly prevent the methyl transfer reaction of M1.HpyAVI. The importance of D29 is preserved because it belongs to the catalytic active site DPPY, but the residue E216 has not been fully investigated even being a conserved amino acid throughout MTases (Figure 4E). E216 is the last residue of β2, which contacts the two hydroxyls of the ribose of AdoMet. Replacement of this residue by alanine completely abolishes the key hydrogen bonds for AdoMet-binding, and very likely blocks the methyl transfer reaction. To confirm this notion, [^3^H]AdoMet radiological assay was applied to quantify the methyl transfer activity of the mutants. As shown in Figure 4D, the result of radiological assay agreed well with the MST measurement. The D29A and E216A mutants showed little or no methyl transfer activity, while other mutants exhibited reduced methyltransferase activity.

As mentioned previously, FXGXG is a conserved AdoMet-binding motif of DNA MTases. We also made mutants of “FMGSG” to alanine for every amino acid, and found that the F195A mutant was insoluble probably due to decreasing the local hydrophobicity upon this mutation. We subsequently investigated the ligand binding affinity and methyl transfer reaction of the other mutants using MST and a radiological assay. We found that G197 played a crucial role in AdoMet-binding, while mutagenesis of M196 and G199 did not influence cofactor binding and catalytic activity (Figure S2A and B). G197 is a conserved residue throughout the DNA MTases, and replacing by alanine at this site likely change the local conformation of cofactor-binding pocket. Mutagenesis on this glycine residue in M.EcoKI or M.EcoP15I also abolished the AdoMet-binding activity [27, 28]. Although mutational study could not tell the role of F195 in ligand binding due to the insolubility of the F195A mutant, structural analysis suggested the importance of this residue in AdoMet-binding. The phenyl ring of F195 forms a perpendicular π-stacking interaction with the purine ring of AdoMet, which stabilizes the orientation of AdoMet bound in the pocket of M1.HpyAVI (Figure S2C). In a separate scenario, mutagenesis of this residue in M.EcoRV has been proven to play an important role in AdoMet binding [29].

### Potential DNA-binding sites

The putative DNA binding region of M1.HpyAVI involves the hairpin loop (residue 101-133), the TRD (residues 136-166), and a highly flexible loop (residues 33-58). The hairpin loop between β6 and β7 strands that carries a conserved HRRY sequence signature in the middle is proposed to insert into the minor groove of the bound DNA [2]. As aforementioned, the TRD of M1.HpyAVI shows striking difference from the other DNA MTases, and the relaxed specificity of substrate recognition may be at least partially attributable to the disordered TRD.

In addition, the highly flexible loop immediately following the DPPY motif in M1.HpyAVI was poorly defined in electron density, exactly like the corresponding loops in the AdoMet-bound structures of M.PvuII, DpnM or M.TaqI that were invisible either [21, 26, 30]. This loop, however, was largely stabilized upon DNA binding, as observed in the protein-DNA complex structures of M.TaqI (PDB ID 2IBS), M.HhaI (PDB ID 1MHT) and M.HaeIII (PDB ID 1DCT). The well-ordered loop in those structures directly contacts the flipping adenine and forms hydrogen bond with neighboring bases [14, 31, 32]. These observations implied that the corresponding loop in other MTases, e.g. M1.HpyAVI, is likely responsible for reducing sequence recognition specificity and thus plays crucial roles in catalysis.

### Key residue for wider spectrum of substrate recognition

**Figure 5:**
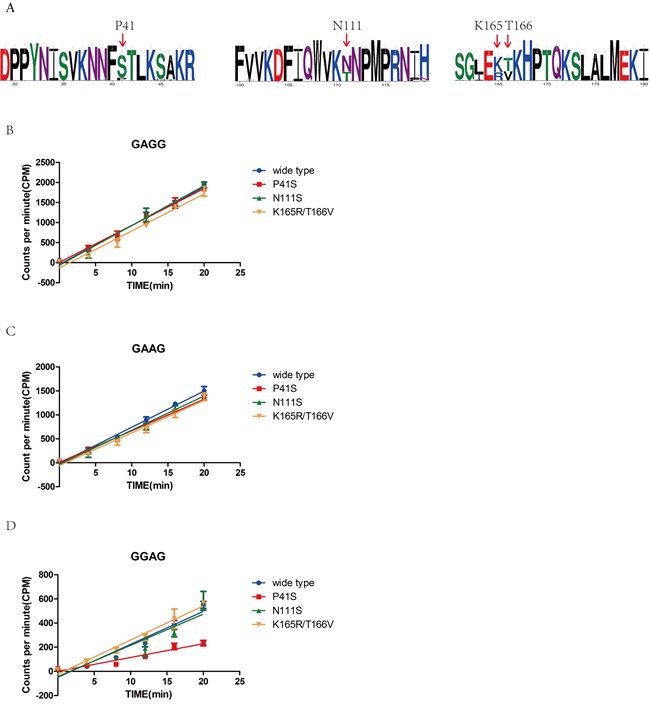
Sequence alignment, structural analysis and radioactive methyl transfer activity define the key residue for wider substrate specificity of M1.HpyAVI **A.** Sequence alignment of M1.HpyAVI from 50 *H. pylori* strains including 26695 revealed several variant residues. Residues P41, N111, K165 and T166 of M1.HpyAVI from strain 26695 were chosen based on structural analysis and sequence alignment (shown in red arrow). Amino-acid conservation is depicted using WebLogo (Crooks et al, 2004). **B.**, **C.**, **D.** Methyl transfer reactions were performed using wt-M1.HpyAVI, M1.HpyAVI-P41S, M1.HpyAVI-N111T, and M1.HpyAVI-K165R T166V, respectively. Radioactivity incorporated into the duplex DNA containing 5′-GAGG-3′, 5′-GAAG-3′ or 5′-GGAG-3′ was quantified by Beckman LS6500 for 10 min. The experiments were repeated for three times and data were corrected by subtraction of the background.

Previous research suggested that M1.HpyAVI from strain 26695 was the first N^6^ adenine MTase that can methylate the adenine of 5′-GAGG-3′/5′-GGAG-3′ or both two adenines of 5′-GAAG-3′, compared with the homologs from other strains that can methylate only one adenine of 5′-GAGG-3′ [20]. To answer why M1.HpyAVI displayed a wider specificity for DNA recognition, we randomly choose fifty of M1.HpyAVI sequences from hundreds of *H. pylori* strains for multiple sequence alignment. Based on sequence comparison and structural analysis, four residues including P41, N111, K165 and T166 were selected and replaced by serine, threonine, threonine and valine, respectively (Figure 5A). Then, a [^3^H]AdoMet radiological assay was applied to quantify the methyl transfer activity of the wide type protein and the mutants. As shown in Figure 5, when the substrate DNA contains 5′-GAGG-3′ or 5′-GAAG-3′, all the mutants showed no apparent difference of methyl transfer activity compared to the wt-M1.HpyAVI; but when the recognition sequence was 5′-GGAG-3′, the methyl transfer activity of the P41S mutant was significantly reduced compared to the wild type M1.HpyAVI.

Our experimental data identified P41 as a key residue determining the recognition of GGAG of M1.HpyAVI. This amino acid locates in the highly flexible loop between residues 33 and 58, which is involved in DNA binding and substrate recognition as shown above. Replacement by serine at this position definitely changes the local conformation and hydrophobicity, and probably some structural properties of the whole loop, which may in turn result in reduced specificity for sequence recognition of the enzyme from strain 26695.

## DISCUSSION

Although the DNA-bound structure of previous investigation on a γ-class N^6^-adenine MTase revealed that the target adenine was rotated out of DNA helix [14], details of the methyl transfer process were still unclear. Additionally, recent studies reported the importance of N^6^-methyladenine in some eukaryotic species, but until now there has not been any N^6^-adenine MTases being identified in eukaryotes. Biochemical and structural characterization of M1.HpyAVI provides a new model for uncovering the methyl transfer mechanism and for investigating the N^6^-methyladenine in eukaryotes.

Oligomeric state of DNA MTases was long accepted as monomer [12], but our study indicated here that M1.HpyAVI exists as a dimer both in crystal and solution. Interestingly, some other β-class DNA exocyclic MTases showed similar oligomeric state in crystal and in solution, indicating that dimer may be the functional state shared by a subgroup of DNA MTases.

The highly flexible region (residues 33-58) and TRD (residues 133-163) of M1.HpyAVI are supposed to interact with DNA at minor and major grooves, respectively. These two structural characteristics may account for the substrate promiscuity of this enzyme. And residue P41 might be a key residue partially determining the substrate spectrum of M1.HpyAVI.

The missing loop between residues 33 and 58 may need DNA binding so as to form a stable conformation, which is similar to the condition of M.TaqI. Crystallization of M1.HpyAVI-DNA complex warrants future investigations, with the purpose of revealing the mechanism behind the wider substrate specificity of this enzyme.

DNA methylation plays an important role in bacterial pathogenicity. DNA adenine methylation was known to regulate the expression of some virulence genes in bacteria including *H.pylori* [17, 18, 33]. Inhibitors of DNA adenine methylation may have a broad antimicrobial action by targeting DNA adenine methyltransferase. As an important biological modification, DNA methylation directly influences bacterial survival. Knockout of M1.HpyAVI largely prevents the growth of *H. pylori* [20]. Importantly, *H. pylori* is involved in 90% of all gastric malignancies [19]. Appropriate antibiotic regimens could successfully cure gastric diseases caused by *H.pylori* infection. However, eradication of *H. pylori* infection remains a big challenge for the significantly increasing prevalence of its resistance to antibiotics [34]. The development of new drugs targeting adenine MTases such as M1.HpyAVI offers a new opportunity for inhibition of *H. pylori* infection. Residues that play crucial roles for catalytic activity like D29 or E216 may influence the *H.pylori* survival. Small molecules targeting these highly conserved residues are likely to emerge less drug resistance.

In summary, the structure of M1.HpyAVI is featured with a disordered TRD and a key residue P41that located in the putative DNA binding region that may associate with the wider substrate specificity. Residues D29 and E216 were identified to play a crucial role in cofactor binding. As the first crystal structure of N^6^-adenine MTase in *H.pylori*, this model may shed light on design of new antibiotics to interfere the growth and pathogenesis of *H.pylori* in human.

## MATERIALS AND METHODS

### Protein expression and purification

The ORF encoding M1.HpyAVI was inserted into the expression plasmid pET22b (Novagen, Massachusetts, USA) to produce a recombinant protein containing a C-terminal His-tag. In order to produce soluble protein, a chaperone plasmid PG-KJE8 (TaKaRa, Dalian, China) was co-expressed with M1.HpyAVI. The recombinant protein was purified with a three-step chromatography protocol using a Ni-NTA affinity column, a HiLoad 16/60 Superdex 200 column and a mono-S HR 5/5 column (1ml) (GE Healthcare, Uppsala, Sweden). Mutants of M1.HpyAVI were generated using the Muta-direct Site-directed Mutagenesis kit (SBS Genetech, Beijing, China) and produced using the same protocol with wide type protein.

### Crystallization and data collection

Crystallization trials were carried out for both the AdoMet-free and AdoMet-bound proteins using the hanging drop vapor diffusion technique. Crystals used for diffraction data collection of the apoprotein were grown under the condition of 1.0 M Bis-Tris, pH 9.0, 1.4 M ammonium tartrate, and the optimal crystallization condition for AdoMet-bound protein was 1.0 M Bis-Tris, pH 6.0, 14% PEG2000, 0.2 M lithium sulfate. X-ray diffraction data were collected at 100 K on beamline BL17U1 at the Shanghai Synchrotron Radiation Facility (SSRF) using an ADSC Quantum 315r CCD detector. All data were indexed, integrated and scaled using the XDS program [35].

### Structure determination and refinement

The structure of ligand-free M1.HpyAVI was determined by means of molecular replacement using the M.MboIIA (PDB ID 1G60) as a search model. Automated structure determination using Phaser [36] gave a solution showing four subunits sitting in the asymmetric unit. The model was refined using the COOT graphics package [37] manually and phenix.refine [38]. The AdoMet-bound structure was determined by means of molecular replacement using the refined model of the apoprotein, and refined in the same way. Statistics from the data collection and structure refinement are summarized in Table 1. All figures representing the M1.HpyAVI structures were generated using the molecular visualization program *PyMol* [39].

### Detection of protein dimerization

The interface information of M1.HpyAVI free form and AdoMet-bound form structures were analyzed using the PDBePISA (Proteins, Interface, Structures and Assemblies) web server.

The protein molecular weight was determined by gel filtration using a FPLC system coupled to a Superdex 75 HR 10 / 30 column. The sizing standard was calibrated using the gel filtration calibration kit LMW (GE Healthcare, Uppsala, Sweden).

### Binding affinity quantification *via* microscale thermophoresis (MST)

Microscale thermophoresis was performed using the NT115 nanotemper technologies. M1.HpyAVI-wt and M1.HpyAVI-mutant proteins were fluorescently labeled using the protein label kit according to manufacturer's protocol. Affinity measurements were performed by using MST buffer (0.05% Tween-20 added as final concentration). A solution of unlabeled AdoMet was serially diluted from 1 mM to 15 nM. Equal volume of 0.8 μM labeled protein was mixed with the AdoMet and loaded into the silica capillaries. This binding curve can directly be fitted with the nonlinear solution of the law of mass action, with the dissociation constant (K_D_) as a result. Measurement was performed at 25°C using 40% LED power and 40%IR-laser power. The dissociation constant was calculated using the Nano-temper Analysis software.

### Radioactive methyltransferase analysis

Several different DNA duplexes containing single site of 5′- GAGG-3′, 5′- GAAG-3′ or 5′-GGAG-3′ were used as substrate for methyl transfer reaction (Table S2). 0.1 μM of enzyme and 2 μM of S-[methyl-^3^H] adenosly methionine (China Isotope and Radiation Corporation, Beijing, China) were incubated at 37°C for 5 min, and then 5 μM of DNA substrate was added to initiate the reaction. Aliquots (20 μl) were taken out at 4-min time intervals and quenched with 2 N HCl. Subsequently, DNA of the mixture was purified using a DNA purification column (TIANGEN, Beijing, China) and the scintillation counting of tritiated DNA was quantified by Beckman LS6500 for 10 min. The background radioactivity was determined by omitting the enzyme from the reaction solution. All the reactions were performed in triplicate.

## SUPPLEMENTARY FIGURES AND TABLES


