# Supplementary material for: Biochemical and structural characterization of a DNA N6-adenine methyltransferase from Helicobacter pylori
Source: Oncotarget. 2016 May 29;7(27):40965–77. doi: 10.18632/oncotarget.9692 (PMC5173035; doi:10.18632/oncotarget.9692)
Supplement: Supplementary file 1 [file oncotarget-07-40965-s001.pdf]

Biochemical and structural characterization of a DNA N^6-adenine methyltransferase from *Helicobacter pylori*

Supplementary Material

Table S1. Residues of the asymmetric unit

	Number of residues	Residues not visible in the structure
M1.HpyAVI Chain A	197	34-57, 160-170
M1.HpyAVI Chain B	185	32-58, 116, 154, 156-172, 232
M1.HpyAVI Chain C	185	32-61, 114, 157-171, 232
M1.HpyAVI Chain D	177	32-60, 111-117, 147, 149, 156-171, 232
M1.HpyAVI-AdoMet Chain A	201	33-58, 169-172, 232
M1.HpyAVI-AdoMet Chain B	194	32-58, 157-164, 230-232
M1.HpyAVI-AdoMet Chain C	204	32-56, 159-160, 232
M1.HpyAVI-AdoMet Chain D	188	32-59, 158-172, 232
M1.HpyAVI-AdoMet Chain E	193	32-58, 158-167, 231-232
M1.HpyAVI-AdoMet Chain F	194	32-58, 159-168, 232
M1.HpyAVI-AdoMet Chain G	200	33-56, 159-163, 230-232
M1.HpyAVI-AdoMet Chain H	193	32-58, 159-168, 231-232

Table S2. Duplex DNA sequences used in the methyl transfer reaction

Duplex	Sequence (5'- to 3')
GAGG-1[a]	CATTTATCAGGGTTATTGTCTCATGAGCGGATACATATTTGAATGTATTTAGAAAAATAAACAAATA<br>GGGGTTCCGCGCACATTTCCCCGAAAAGTGCCACCTGACGTCTAAGAAACCATTATTATCATGACAT<br>TAACCTATAAAAATAGGCGTATCAC**GAGG**CCCTTTCGTC
GAGG-2[a]	CTATTACGCCAGCTGGCGAAAGGGGGATGTGCTGCAAGGCGATTAAGTTGGGTAACGCCAGGGTTTT<br>CCCAGTCACGACGTTGTAAAACGACGGCCAGTGAATTCGAGCTCGGTACCCGGGGAT**CCTC**TAGAGT<br>CGACCTGCAGGCATGCAAGCTTGGCGTAATCATGGTCATAGCTGTTTCCTGTGTGAAATTGTTATCCG
GAAG-1[b]	TAGAGTCGACCTGCAGGCATGCAAGCTTGGCGTAATCATGGTCATAGCTGTTTCCTGTGTGAAATTGT<br>TATCCGCTCACAATTCCACACAACATACGAGCCG**GAAG**CATAAAGTGTAAAGCCTGGGGTGCCTAAT<br>GAGTGAGCTAACTCACATTAATTGCGTTGCGCTCACTGCCCGCTTTCCAGTCGGGAAACCTGTCGTGC
GAAG-2[b]	CGTTGTCA**GAAG**TAAGTTGGCCGCAGTGTTATCACTCATGGTTATGGCAGCACTGCATAATTCTCTTA<br>CTGTCATGCCATCCGTAAGATGCTTTTCTGTGACTGGTGAGTACTCAACCAAGTCATTCTGAGAATAG<br>TGTATGCGGCGACCGAGTTGCTCTTGCCCGGCGTCAATACGGGATAATACCGCGCCACATAGCAGAA
GGAG-1[c]	GTGGCGCTTTCTCAATGCTCACGCTGTAGGTATCTCAGTTCGGTGTAGGTCGTTCG**CTCC**AAGCTGGG<br>CTGTGTGCACGAACCCCCCGTTCAGCCCGACCGCTGCGCCTTATCCGGTAACTATCGTCTTGAGTCCA<br>ACCCGGTAAGACACGACTTATCGCCACTGGCAGCAGCCACTGGTAACAGGATTAGCAGAG
GGAG-2[c]	AGAAATTCTTTCATCTCTTTCGGCGGCTGGTAACCCGGAACAAGTGTGCCATTGCTCAGCACAACTGC<br>C**GGAG**TACCGCTAACGCCAAGCTGGACGCCAAGTGCGTAATGGTCGGCAATATCCACGTCGCAACT<br>GGCTGGTGCGACGCTTTTACCTGCCATCACATCATCAAACGCTTTGTTTTTATCTTTCGCACACCAGA
N-Control-1[d]	TAAGTTGGCCGCAGTGTTATCACTCATGGTTATGGCAGCACTGCATAATTCTCTTACTGTCATGCCAT<br>CCGTAAGATGCTTTTCTGTGACTGGTGAGTACTCAACCAAGTCATTCTGAGAATAGTGTATGCGGCG<br>ACCGAGTTGCTCTTGCCCGGCGTCAATACGGGATAATACCGCGCCACATAGCAGAACTTTAAAAGTG
N-Control-2[d]	ATTACAGAAACGGCTTTTTCAAAAATATGGTATTGATAATCCTGATATGAATAAATTGCAGTTTCATT<br>TGATGCTCGATGAGTTTTTCTAAGAATTAATTCATGAGCGGATACATATTTGAATGTATTTAGAAAAA<br>TAAACAAATAGGGGTTCCGCGCACATTTCCCCGAAAAGTGCCACCTGAAATTGTAAACGTTAATATT

[a] DNA substrates that contain 5'-GAGG-3' sequence; [b] DNA substrates that contain 5'-GAAG-3' sequence; [c] DNA substrates that contain 5'-GGAG-3' sequence; [d] DNA substrates that used as negative control.

```
BAD AVG GOOD

M1.HpyAVI       MIQI--------------------------------------YHADAFEIIKDFYQQNLKVDAIITDPP 31
M.MboIIA        MLEI-----------------------------------NKIHQMNCFDFLDQVE--NKSVQLAVIDPP 32
M.RsrI          MANRSHHNAGHRAMNALRKSGQKHSSESQLGSSEIGTTRHVYDVCDCLDTLAKLP--DDSVQLIICDPP 67
TTHA0409        MRSFPKA-------------KEAFS-----EGEKVSFGVHRLHVGDAREVLASFP--EASVHLVVTSPP 49
cons            * .                                          :. : : ..   : .*.  : .**

M1.HpyAVI       YNISVKNNFPTLKSAKRQGIDFGEWDKNFKLLE----WIARYAPLVNPNGCMVIFCSYR---------- 86
M.MboIIA        YNLSKADW-DS-------------FDSHNEFLAFTYRWIDKVLDKLDKDGSLYIFNTPF---------- 77
M.RsrI          YNIMLADW-----------------DDHMDYIGWAKRWLAEAERVLSPTGSIAIFGGLQYQGEAG--SG 117
TTHA0409        YWTLKRYE-DT-------PGQLGHIEDYEAFLDELDRVWREVFRLLVPGGRLVIVVGDVAVARRRFGRH 110

cons            *                        :.    :        .    :   * : *.

M1.HpyAVI       -FIS---YIADFLEENGFVVKDFIQWVKNNPMPRNIHRR--YVQDTEFALWAVKKKAKWVFNKP-KNEK 148
M.MboIIA        -NCA---FICQYLVSKGMIFQNWITWDKRDGMG-SAKRR--FSTGQETILFFSKSKN-HTFNYDEVRVP 138
M.RsrI          DLIS---IISHMRQNSKMLLANLIIWNYPNGM--SAQRF--FANRHEEIAWFAKTKK-YFFDLDAVREP 178
TTHA0409        LVFPLHADIQVRCRKLGFDNLNPIIWHKHTNASLEVEGRGVFLGKPYEPGAIIKTEI-EYILMQRKPGG 178
cons               .    *     .  :   : * *        . .    :           *.:    :

M1.HpyAVI       YL---------------------------RP--LILK-----SPVVSG--LEKTKHPTQKSLALMEKII 181
M.MboIIA        YESTDRIKHASEKGILKNGKRWFPNPNGRLCGEVWHFSSQRHKEKVNGKTV-KLTHITPKPRDLIERII 206
M.RsrI          YDEETKAAYMKDKRLNPES-----VEKGRNPTNVWRM------SRLNGNSLERVGHPTQKPAAVIERLV 236
TTHA0409        YRKPTQEQ----------------REKSRLPKEDFHRFFRQIWDDIPG--ESTKDHPAPFPLELAERLV 229
cons            *                                            : *       * :  .  : *:::

M1.HpyAVI       SIHTNPNDIVLDPFMGSGTTGLACKNLERNFIGIESEKEYFQTAKKRLNLF------------------ 232
M.MboIIA        RASSNPNDLVLDCFMGSGTTAIVAKKLGRNFIGCDMNAEYVNQANFVLNQL------------EIN--- 260
M.RsrI          RALSHPGSTVLDFFAGSGVTARVAIQEGRNSICTDAAPVFKEYYQKQLTFLQDDGLIDKARSYEIVEGA 305
TTHA0409        RMFSFVGDVVLDPFAGTGTTLIAAARWGRRALGVELVPRYAQLAKERFARE-V-----PGFSLEVLDGA 292
cons               :  .. *** * *:*.*  .. .  *. :  :    : :  :  :

M1.HpyAVI       --------------
M.MboIIA        --------------
M.RsrI          ANFGAALQRGDVAS 319
TTHA0409        THPRR--------- 297
cons
```

Figure S1. Multiple sequence alignment among M1.HpyAVI and other β-class N^6 adenine or N^4 cytosine MTases.

The highly flexible region of M1.HpyAVI (residues 33-58) is longer while the TRD of M1.HpyAVI (residues 136-166) is shorter in length, compared with the other three sequences. Residues coloring from blue through yellow to red indicate the similarity among sequences. The multiple sequence alignment was performed using T-coffee web server.

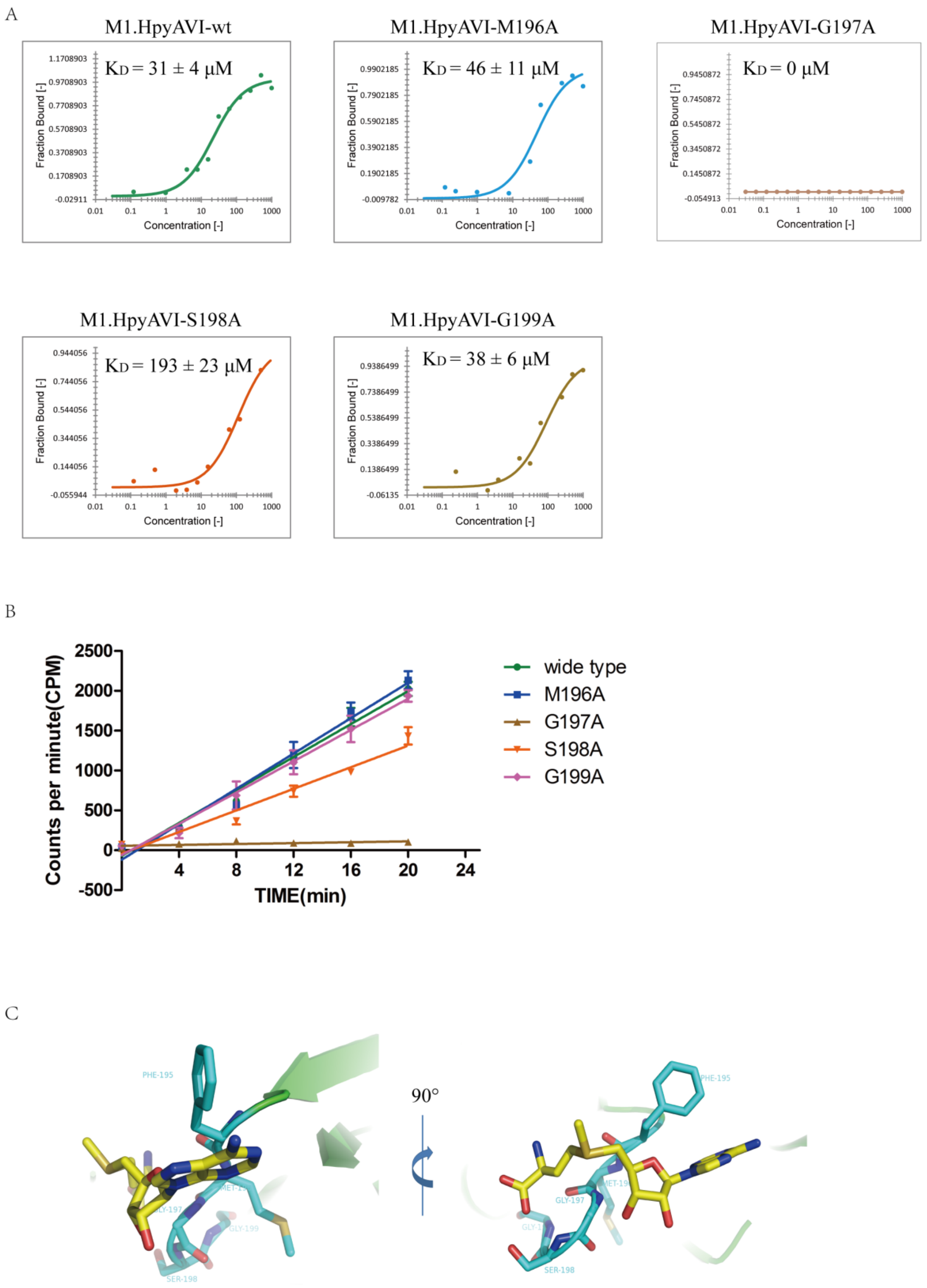

Figure S2. Structural and biochemical analyses of the conserved domain "FMGSG" for AdoMet binding

A. Cofactor binding affinity of wt-/mutants M1.HpyAVI proteins analyzed by microscale thermophoresis (MST). The binding affinity was determined between

fluorescently labelled M1.HpyAVI protein and unlabeled AdoMet. The bound fraction is shown on the *y*-axis against the protein concentration. AdoMet (15 nM to 1 mM) was titrated into a fixed concentration of M1.HpyAVI wt/mutant protein (800 nM). The dissociation constant (K_D) is yielded according to the law of mass action from the isotherm derived of the raw data: M1.HpyAVI-wt: 31 ± 4 μM; M1.HpyAVI-M196A : 46 ± 11 μM; M1.HpyAVI-G197A : 0 μM; M1.HpyAVI-S198A : 193 ± 23 μM; M1.HpyAVI-G199A : 38 ± 6 μM. Standard deviation for three replicates is indicated. Measurements were made with 40% LED and 40% laser power at 25°C. **B.** Methyl transfer activity of wide type protein and the mutants were quantified using radioactive assay. [^{3}H]-methyl transferred to duplex DNA containing 5'-GAGG-3' was quantified by Beckman LS6500 for 10 min. Experiments were repeated for three times and data were corrected by subtraction of the background. **C.** Interaction of F195 and AdoMet in the cofactor-bound structure of M1.HpyAVI.

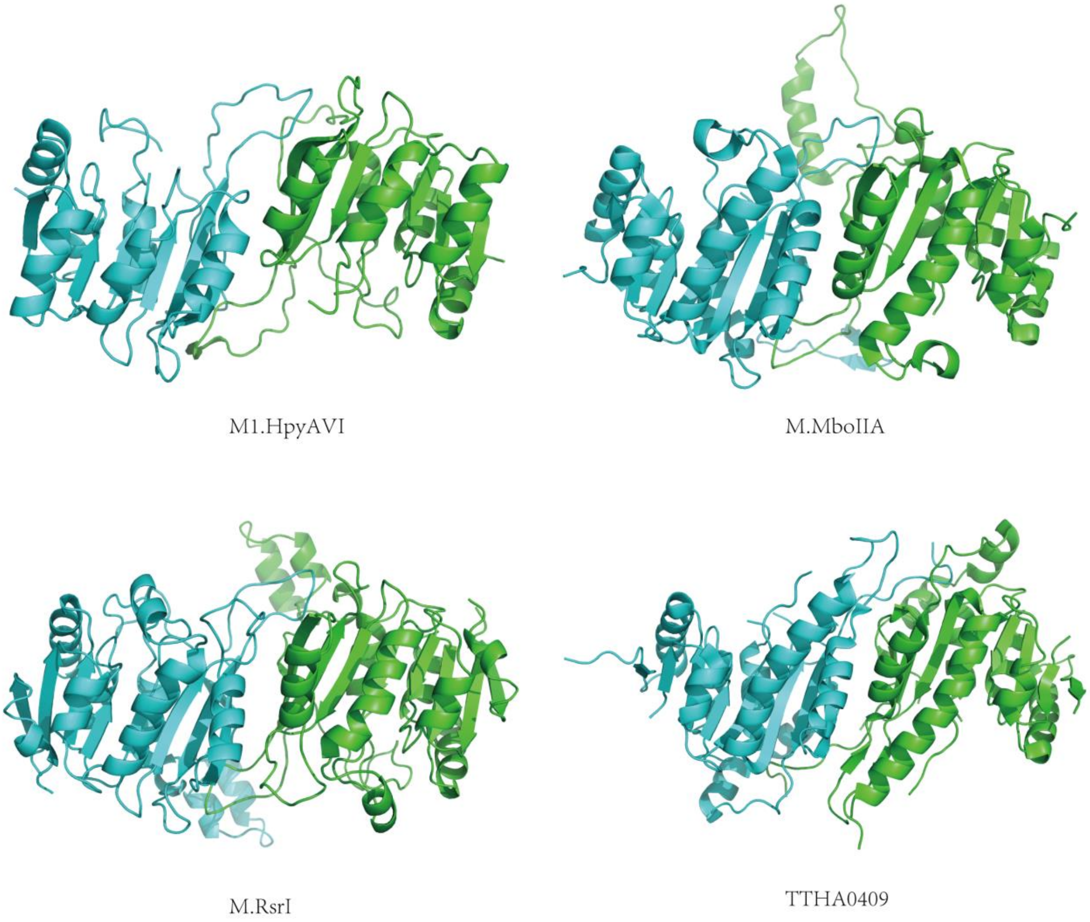

Figure S3. Dimer organization of M1.HpyAVI, M.MboIIA, M.RsrI and TTHA0409

The two monomers are marked in green and blue. The pictures were generated using *PyMol*.

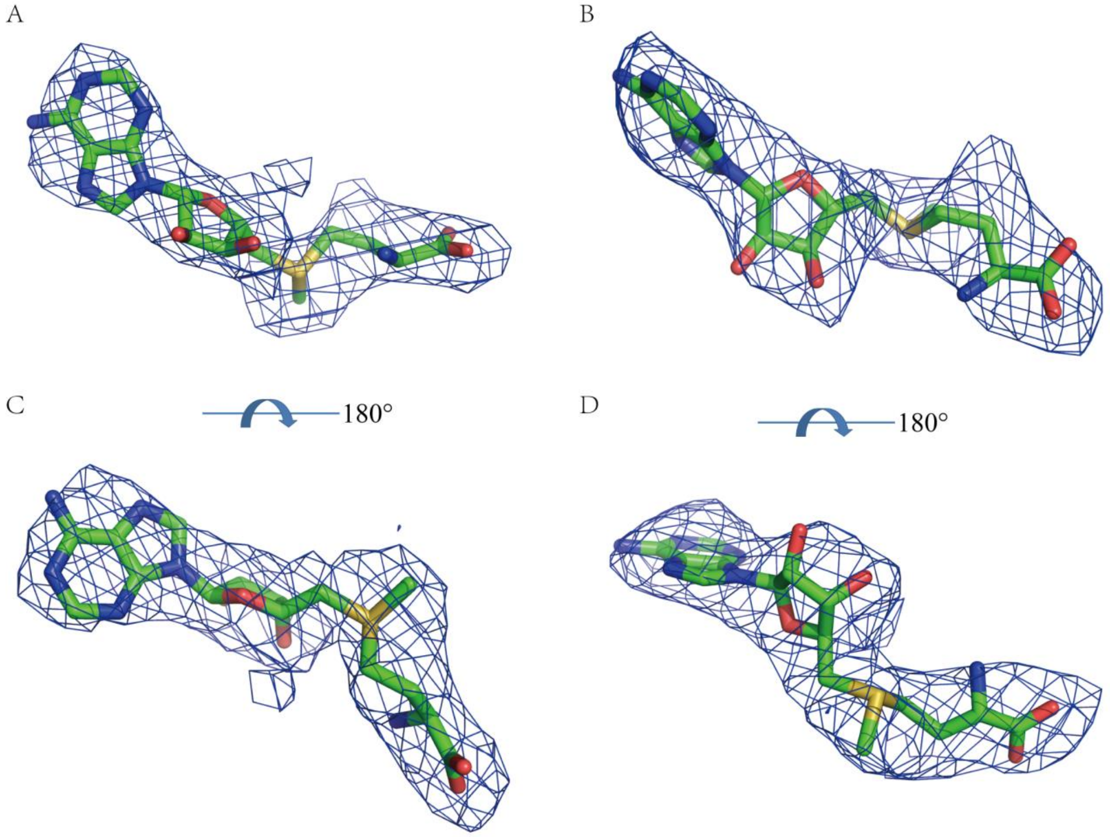

Figure S4.The *Fo-Fc* omit map of the AdoMet in the M1.HpyAVI binary complex structure.

The pictures were generated using *PyMol*.
